# Computationally Enhanced, Haemodynamic Case Study of Neointimal Hyperplasia Development in a Dialysis Access Fistula

**DOI:** 10.31083/j.rcm2501035

**Published:** 2024-01-22

**Authors:** Matthew Bartlett, Mirko Bonfanti, Vanessa Diaz-Zuccarini, Janice Tsui

**Affiliations:** ^1^Division of Surgery & Interventional Science, University College London, Royal Free Campus, NW3 2QG London, UK; ^2^Department of Vascular Surgery, Royal Free London NHS Foundation Trust, NW3 2QG London, UK; ^3^Department of Mechanical Engineering, University College London, WC1E 7JE London, UK; ^4^Wellcome/EPSRC Centre for Interventional and Surgical Sciences (WEISS), Department of Medical Physics and Biomedical Engineering, University College London, W1W 7TS London, UK

**Keywords:** CFD, ultrasound, dialysis access, haemodynamics, turbulence intensity

## Abstract

**Background:**

Oscillatory wall shear stress and related metrics have been identified as potential predictors of dialysis access outcomes; however, the absence of a simple non-invasive method for measuring these haemodynamic forces has been prohibitive to their adoption into routine clinical practice. We present a computationally enhanced, single patient case study, offering a unique insight into the haemodynamic environment surrounding the development of flow limiting neointimal hyperplasia within the efferent vein of a previously functional arteriovenous fistula (AVF).

**Methods:**

Computational fluid dynamics (CFD) simulations were used to create a quantitative map of oscillatory shear stress as well as enabling visualisation of streamline patterns within the AVF. CFD data was compared to ultrasound-based turbulence quantification and examined alongside structural and functional changes in the access site over time.

**Results:**

This work further supports the notion that flow limiting neointimal hyperplasia development in vascular access fistulae, occurs in response to oscillatory wall shear stress, and provides proof of concept for the idea that non-invasive ultrasound turbulence quantification tools could play a role in predicting vascular access outcomes.

**Conclusions:**

In addition to providing insight into the haemodynamic environment surrounding the development of flow limiting neointimal hyperplasia, we hope that this paper will promote discussion and further thinking about how our learnings from *in-silico* studies can be incorporated into clinical practice through novel uses of existing diagnostic tools.

## 1. Introduction

It is estimated that 80% of thrombotic occlusions in dialysis access arteriovenous fistula (AVF) occur secondary to neo-intimal hyperplasia (NIH) stenosis formation [1]. The processes leading to accelerated NIH development in the dialysis population are complex and multi-faceted, with influencing factors including endothelial injury from surgery and repeated venipuncture [2], the impact of chronic uraemia [3], and response to the altered haemodynamics of the AVF circuit [4]. The presence of NIH prior to AVF formation is not a reliable predictor of outcomes [5], suggesting that uraemia and other biochemical changes associated with chronic kidney disease (CKD) are not the primary cause of access failure. Although the anastomosis, and regular needling sites are both common regions for thrombus formation, the majority of flow limiting lesions resulting from NIH are situated in the juxta-anastomotic venous swing segment and the cephalic arch [6], neither of which are regular puncture sites. This suggests that it is the altered venous haemodynamics which is primarily responsible for the accelerated NIH proliferation rather than direct endothelial trauma.

In recent years computational fluid dynamics (CFD) has enabled us to gain a better understanding of the nature of these haemodynamic stresses, providing information on flow patterns, with a degree of spatial and temporal resolution, which is not possible using conventional diagnostic imaging modalities. However, the time and expertise required to run these simulations, along with the considerable computational expense remain a barrier to widespread translation into the clinical setting.

The aim of this CFD-enhanced case study was to observe the relationship between ultrasound-based turbulence measurements, and the oscillatory shear stress patterns, which can only be quantified with the use of CFD, with the hope of developing techniques for translating the wealth of knowledge obtained from CFD based research, into clinical diagnostics and surveillance.

## 2. Methods

### 2.1 Patient Recruitment and Follow Up

A single patient with a fully matured, functioning radio-cephalic AVF (RCAVF) was consented for this study.

Inclusion Criteria: Fully matured upper limb AVF being used for regular haemodialysis (HD) with 2 needles. 18 years of age or older and able to provide informed consent.

Exclusion Criteria: Previous surgical or endovascular salvage of the AVF. Previous central venous catheter on the side of the AVF. Repeated episodes of elevated venous pressures or high recirculation rates on HD in the previous 3 months.

At the time of recruitment, the patient had been using the AVF for regular HD for a period of 3 months. Following an initial ultrasound examination and magnetic resonance imaging (MRI) scan, the patient was followed up with ultrasound surveillance over the next 8 months allowing for structural and functional changes in the vascular anatomy to be monitored and compared with the initial ultrasound derived turbulence intensity ratio (USTIR) and shear stress measurements.

The ethical considerations surrounding the use of radiological contrast agents in patients with end stage renal failure (ESRF) meant that axial imaging was limited to a single timepoint. Computed tomography angiography (CTA) contrast agents are nephrotoxic and risk depleting any residual kidney function. It was decided that imaging would be obtained from contrast enhanced magnetic resonance angiography (CEMRA). The rapid change in flow direction at the anastomosis means that resolution of non-contrast magnetic resonance angiography (MRA) can be limited in this region. Due to risks of nephrogenic systemic fibrosis (NSF), gadolinium based contrast agents (GBCA) should be used with caution in patients with ESRF [7], however following a literature review and full risk assessment we were unable to identify any reported cases of NSF resulting from an isolated administration of Dotarem® (gadoteric acid).

The original timeline for the planned surveillance scans was adapted to match the patient’s clinical requirements. Table 1 shows the time points used for data collection.

**Table 1.** **Assessment Timeline**.

Months from AVF Formation	Event	Flow Volume	Outcome
0	Surgery		
2	US Assessment	~700 mL/min	Use for dialysis (2 needles)
5	US & CEMRA	~700 mL/min	CFD Model created
10	US Assessment	~550 mL/min	Continue surveillance
13	US Assessment	~250 mL/min	Referral for fistulaplasty

CFD, computational fluid dynamics; CEMRA, contrast enhanced magnetic resonance angiography; AVF, arteriovenous fistula.

### 2.2 Ultrasound Protocol

We have previously developed a standardised methodology, using a diagnostic ultrasound scanner, to quantify the degree of turbulence at a selected point within an AVF, and have demonstrated a correlation between elevated USTIR and NIH development in the efferent vein of maturing AVF [8].

Cardiac gated Doppler frequency spectra were recorded as audio data in a 24 bit .wav format, with a sample rate of 44.1 kHz. Recordings of 25 cardiac cycles were obtained from each acquisition zone, Fourier transforms were performed, and an ensemble averaging technique was used to isolate the non-cyclical components of the resulting spectrograms. The isolated components of the frequency spectra, assumed to correspond to random fluctuations due to turbulence, were averaged over the cardiac cycle and then normalised by the average frequency shift at each location to obtain the USTIR. We have previously detailed the data collection and analysis protocol, and validated this technique in patients with surgically created AVF [8].

USTIR was calculated at 1 cm intervals along the radial artery and the cephalic vein. The complete AVF circuit was also assessed for clinical functionality and the presence of any hamodynamically significant stenotic disease. A moderate stenosis was identified in the radial artery, which was also evident on the CEMRA and the CFD mesh, however it did not appear to significantly alter flow waveforms at the level of the anastomosis. Despite the irregularities in the luminal diameter of the cephalic vein, and the presence of mild NIH at the anastomosis there were no flow limiting lesions identified throughout the efferent veins (anastomosis to subclavian vein).

Follow up ultrasound scans were conducted to assess for structural remodelling of the vessels and NIH development, along with changes in access flow over time. Where detected on ultrasound, NIH was graded as minor, moderate or significant, based on sonographic appearance and peak systolic flow velocities (PSV), as defined in Table 2.

**Table 2.** **Sonographic grading of neointimal hyperplasia (NIH)**.

Grading of NIH	Sonographic Definition
None	Smooth, well defined intima-media complex with no visible NIH or plaque
Minor	NIH visible on B-mode ultrasound, no measurable haemodynamic effect
Moderate	Stenotic lesion resulting in an increase in PSV of <2×
Significant	Stenotic lesion resulting in an increase in PSV of >2×

NIH, neointimal hyperplasia; PSV, peak systolic flow velocities.

### 2.3 Development of the CFD Model 

The geometry for the simulation was based on the CEMRA of the upper limb. The data were exported in Digital Imaging and Communications in Medicine (DICOM) format and axial image slices were loaded into ${\mathrm{Simpleware}}^{\text{TM}}$ Scan IP (Synopsys Inc, Irvine, CA, USA).

A 3D volume was generated from the segmented images and a smoothing algorithm was applied to compensate for the image artefact introduced by the discrete slice thickness of each image. The smoothed volumetric model was then imported into Ansys MESH (Ansys Inc, Canonsburg, PA, USA) for initial meshing and refinement. Prior to meshing comparisons were made between luminal diameters of the original CEMRA images and the imported fluid volume at selected regions (anastomosis, max diameters and min diameters), and were found to be comparable within the limits of the resolution of the original scan. The only exception to this was the moderate narrowing in the inflow artery, which was slightly exaggerated on the smoothed model, however given that this region of the model was not our main area of interest it was decided that the improvements gained by smoothing the model justified the introduction of this minor discrepancy in the geometry. An unstructured mesh consisting of tetrahedral elements was created, and wedge shaped elements were used to generate inflation layers to ensure optimum resolution in our main region of interest, adjacent to the vessel walls.

The mesh was refined until successive versions showed less than 10% variation in the average oscillatory shear index (OSI) values for each vessel segment, ensuring good agreement on both distribution and magnitude of oscillatory shear between successive simulations. In order to achieve this, considerable refinement was required and the final mesh consisted of approximately 3.5 million elements, and incorporated 10 inflation layers with a minimum height of 0.025 mm and a total thickness of 0.65 mm.

#### 2.3.1 Boundary Conditions

The boundary conditions for the simulation were based on the Doppler data obtained from the initial ultrasound examination, which was conducted on the same day as the CEMRA. Due to the presence of reverse flow in the radial artery distal to the anastomosis, the simulation required 2 independent input waveforms, both of which were generated from the peak velocity envelope of the Doppler waveform, obtained over a single cardiac cycle. Time dependent inputs were then generated based on the assumption of parabolic flow profiles, with the centreline velocity derived from the peak velocity envelope of the ultrasound waveform. The resulting flow profiles were validated against the time averaged mean velocity and estimated flow volume measurements generated by the ultrasound system at the time of the initial scan.

The exit conditions were based on the flow split between the main cephalic vein and the accessory branch, as measured on ultrasound. This represented a split of 98% of the flow up the main cephalic vein with the remaining 2% being diverted up the accessory branch. Smaller branches visible on the CEMRI were excluded from the model as those detected on ultrasound diverted less than 1% of the total flow from the cephalic vein and were therefore considered of negligible effect compared to other assumptions incorporated into the model.

#### 2.3.2 Fluid Properties

The simulation was run using both Newtonian and non-Newtonian fluid models. The Newtonian model assumed blood density of 1060 ${\mathrm{kg/m}}^{3}$ and viscosity of 4 × ${\mathrm{10}}^{-3}$ Pa.s. The non-Newtonian simulation was based on the Carreau-Yasuda viscosity model, using parameters from literature, originally derived from experimental data [9], and further validated in models of end-side anastomoses similar to the geometry we present here [10]: Viscosity at zero shear = 22 × ${\mathrm{10}}^{-3}$ Pa.s, Viscosity at infinite shear = 2.2 × ${\mathrm{10}}^{-3}$ Pa.s, Relaxation time = 0.11 s, Power Index = 0.392, Yasuda exponent = 0.6444.

Estimations of Reynold’s number (Re), based on values obtained from the ultrasound scan correspond to laminar flow conditions (Re ≈ 1200), with a tendency towards transitional flow for peak velocity values (Re ≈ 2200). Despite this, it is evident from the degree of spectral broadening present in the Doppler waveforms, that flow disturbances resulting from the geometry, are present in the vein throughout the cardiac cycle. The k-omega shear stress transport model (k-ω SST), is validated for low Re, and is generally considered to provide good accuracy for estimating boundary layer conditions close to the walls, however it can result in an over-estimation of turbulence levels in regions of high acceleration or flow stagnation [11]. Although the k-ω SST model is well suited to the flow conditions in our main regions of interest, we know that flow separation and stagnation is common around the anastomosis of AVF, which may result in potential discrepancies in this region. For this reason, the simulation was run using both laminar and k-ω SST models.

#### 2.3.3 Assumptions and Model Limitations 

Rigid, non-slip walls were defined throughout the model.

We acknowledge that the assumption of rigid walls is not representative of true physiological conditions, however this assumption has previously been tested in CFD simulations of dialysis AVF, and despite influencing the magnitude of shear stress indices, distribution patterns were shown to be consistent with more complex and computationally expensive models [12].

### 2.4 Shear Stress Metrics

The magnitude and distribution of a range of haemodynamic parameters were obtained from the results of the CFD simulation. Our primary interest was in identifying regions of oscillatory flow, quantified using the OSI, which has previously been linked to NIH formation in vascular access AVF [13], and intuitively was the most likely metric to map to regions of high USTIR.

We know that when an AVF is formed the act of bypassing the distal capillary bed results in a sudden decrease in distal resistance and a corresponding increase in flow. This leads to an elevation in wall shear stress (WSS), triggering a vasodilatory response, which plays a key role in the vascular remodelling observed following AVF formation. Due to the pulsatile nature of blood flow WSS is continuously variable over the cardiac cycle, for this reason time averaged values of WSS (TAWSS) are often quoted rather than absolute maximum or minimum values.

$$T\,A\,W\,S\,S=\frac{1}{T}\,\int_{0}^{T}\left|W\,S\,S\right|\,𝑑t$$

The disturbed nature of the flow around the anastomosis of a native AVF means that the direction and magnitude of WSS is continuously changing throughout the cardiac cycle. Ku *et al*. [14] devised the OSI to quantify the deviation of WSS from the average direction.

$$O\,S\,I=0.5\,\left[1-\frac{\left|\int_{0}^{T}W\,S\,S\,𝑑t\right|}{\int_{0}^{T}\left|W\,S\,S\right|\,𝑑t}\right]$$

It has been previously shown that both low TAWSS and high OSI may be contributing factors to NIH formation and resultant vascular access dysfunction [13, 15, 16].

Another quantitative metric of shear forces, commonly encountered in literature in relation to AVF failure, is relative residence time (RRT), which combines the TAWSS and OSI, and is defined as [17]

$$R\,R\,T={\left[\left(1-2\cdot O\,S\,I\right)\cdot T\,A\,W\,S\,S\right]}^{-1}$$

A variation of RRT is Highly Oscillatory Low MagnitudE Shear (HOLMES), which is equal to half of the reciprocal of RRT. HOLMES has the advantage of providing a linear index, which is more conceptually intuitive, providing a value corresponding to TAWSS, which continues to drop in the presence of high OSI [18]. Holmes has previously been shown to be a predictor of NIH growth in venous bypass conduits exposed to arterial flow in the lower limbs [19].

H⁢O⁢L⁢M⁢E⁢S=T⁢A⁢W⁢S⁢S⋅(0.5-O⁢S⁢I)

**Fig. 1.**
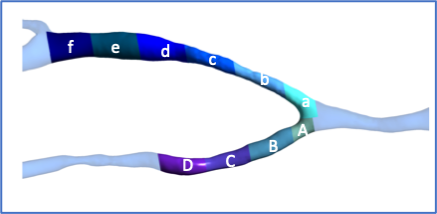
**Observation sites used for analysis**. Radial Artery: A, B, C, D. Cephalic Vein: a, b, c, d, e, f.

The CFD model was divided into segments, 1 cm in length, corresponding to the USTIR observation sites (Fig. 1). The shear stress parameters were averaged over the vessel walls for each of the segments allowing for magnitude and distribution of OSI and HOLMES to be compared with our ultrasound-based predictions of turbulent flow regions. We also recorded the maximum OSI and minimum HOLMES values for each vessel segment.

## 3. Results

### 3.1 Comparison of 3 CFD Simulations 

**Fig. 2.**
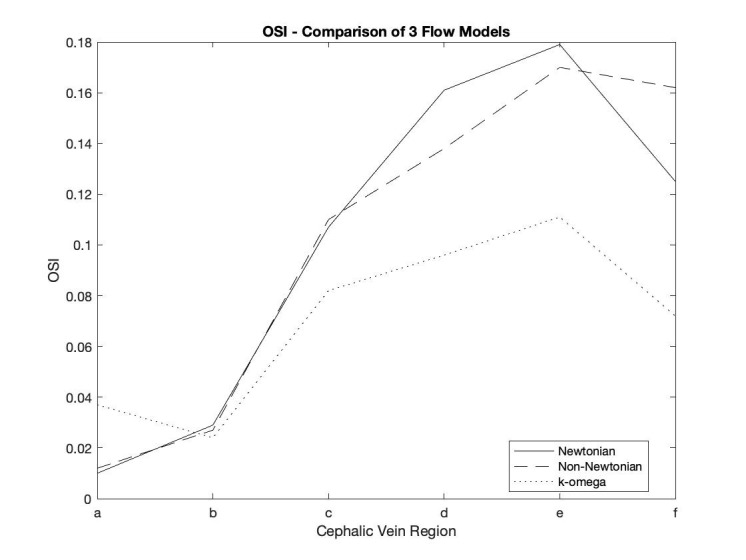
**Comparison of OSI in the cephalic vein based on 3 different simulations**. OSI, oscillatory shear index.

Different fluid properties and turbulence models were compared, whilst the patient specific mesh and boundary conditions were identical for all 3 simulations. Fig. 2 shows the average OSI in the pre-defined regions of the cephalic vein, as produced by each of the 3 simulations.

The Newtonian and non-Newtonian fluid models provide very similar results for the low OSI regions, however there is some degree of discrepancy between the 2 models in zones d, e and f, which correspond the regions with the highest OSI. Given that the assumption of approximated Newtonian behaviour of blood, is only valid for laminar flow in large vessels, these results suggest that the addition of the non-Newtonian model is necessary to accurately quantify OSI in regions of disturbed flow. It should be noted however, that despite the quantitative differences between these 2 models the OSI distribution patterns remain similar, with both simulations successfully differentiating between regions of high and low OSI.

The addition of the k-ω SST model resulted in a reduction in the OSI in all regions of the cephalic vein, except for zone a; the juxta-anastomotic segment, where calculations of OSI was significantly higher than the 2 simulations based on the laminar flow model. The k-ω SST model is known to over-estimate turbulence in regions of rapidly accelerating, pulsatile flow, which may explain this discrepancy. All other zones showed very similar distributions of OSI to the non-Newtonian, laminar flow model, however the magnitude was reduced across the entire vessel.

Due to the limitations in validating the k-ω SST model, it was decided that the non-Newtonian, laminar flow simulation would be used for the remainder of the analysis. Variations in the magnitude of OSI on the laminar and non-laminar simulations are not of great significance as we do not have a range of normal, or expected values for comparison, so for the purposes of this case study it is the distribution of OSI that is of interest.

**Fig. 3.**
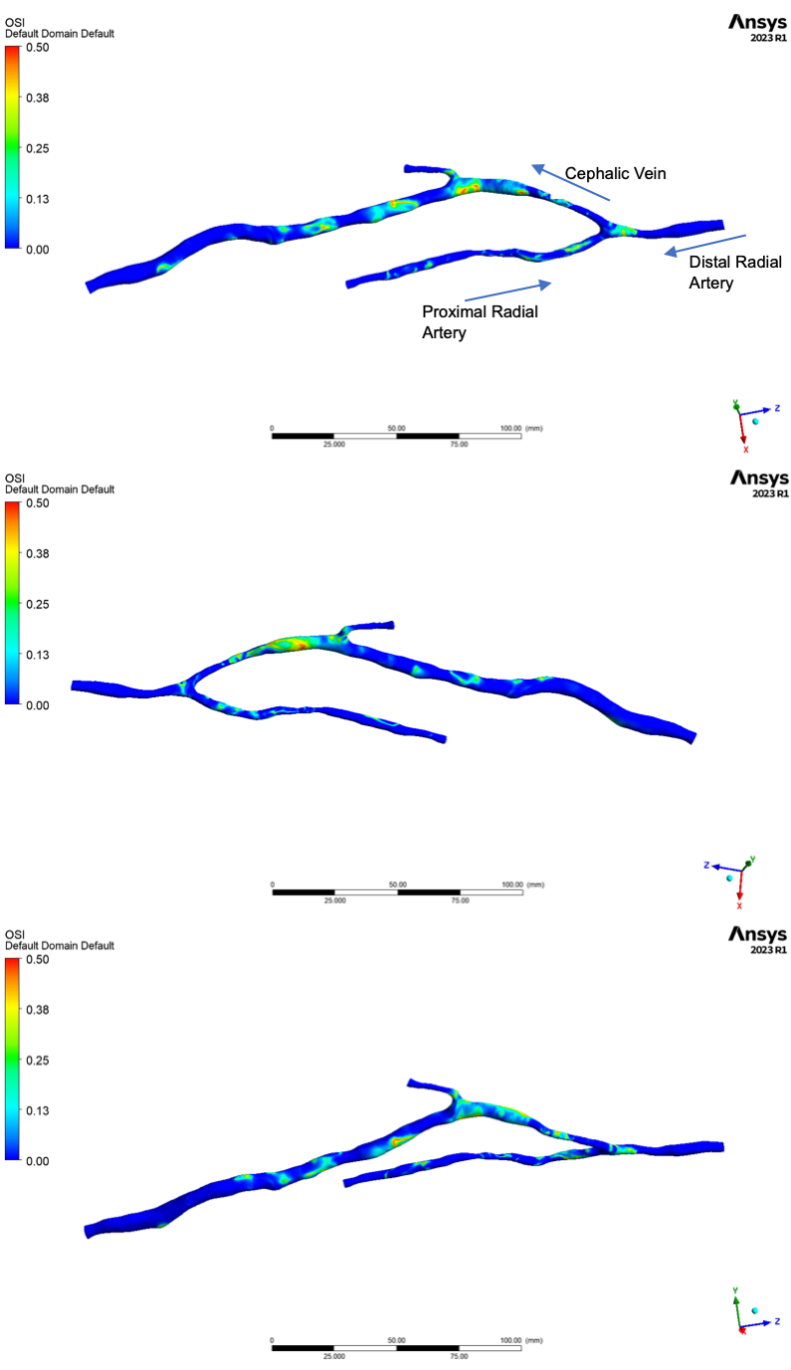
**Distribution of oscillatory shear index (OSI) as calculated by the computational fluid dynamics (CFD) simulation**.

**Fig. 4.**
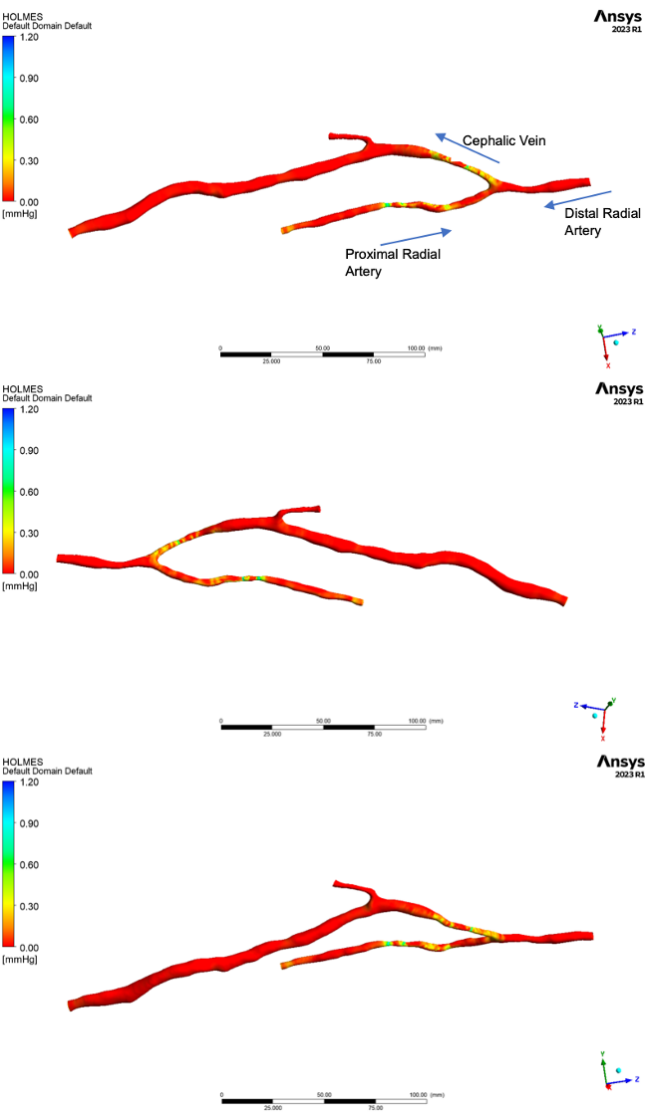
**Distribution of Highly Oscillatory Low MagnitudE Shear (HOLMES) as calculated by the computational fluid dynamics (CFD) simulation**.

Figs. 3,4 show the distribution of OSI and HOLMES respectively, across 3 different projections of the 3D geometry. The colour map used in Fig. 3 to represent the values of OSI throughout the modelled anatomy provides useful visual guide to both localised OSI maximums, as well as regions with globally elevated oscillatory shear. In the artery slight elevations are seen distal to regions of vessel curvature, and also at the distal edge of the anastomosis, where retrograde flow from the hand mixes with the antegrade flow, which provides the majority of the inflow.

Distribution patterns in the vein appear far more complex, with elevated OSI spread over larger regions with greater maximum values. Interestingly the juxta-anastomotic segment of the vein (zones a and b on Fig. 1), show relatively low OSI, with values increasingly as flow travels along the vein into the dilated segment (zones d, e and f on Fig. 1).

The colour map used in Fig. 4 is less intuitive due to the wide scale required to capture localised regions of high HOLMES. This is problematic as it means that the regions of low HOLMES, in which we are interested are poorly represented by the chosen scale. Although the scale is easily adjusted to allow us to better highlight these regions, the presented images provide a useful insight into why metrics which incorporate TAWSS may prove less reliable in the high flow environment typical of AVF. It is clear from Fig. 4, that the distribution of HOLMES in both the venous and arterial sections of the model, is heavily dependent on the vessel diameter. We know that under laminar flow conditions WSS is proportional to flow velocity, and therefore vessel diameter. AVF typically exhibit high velocity flow, and commonly have irregular luminal diameters, resulting in large differences in TAWSS throughout the flow circuit.

**Fig. 5.**
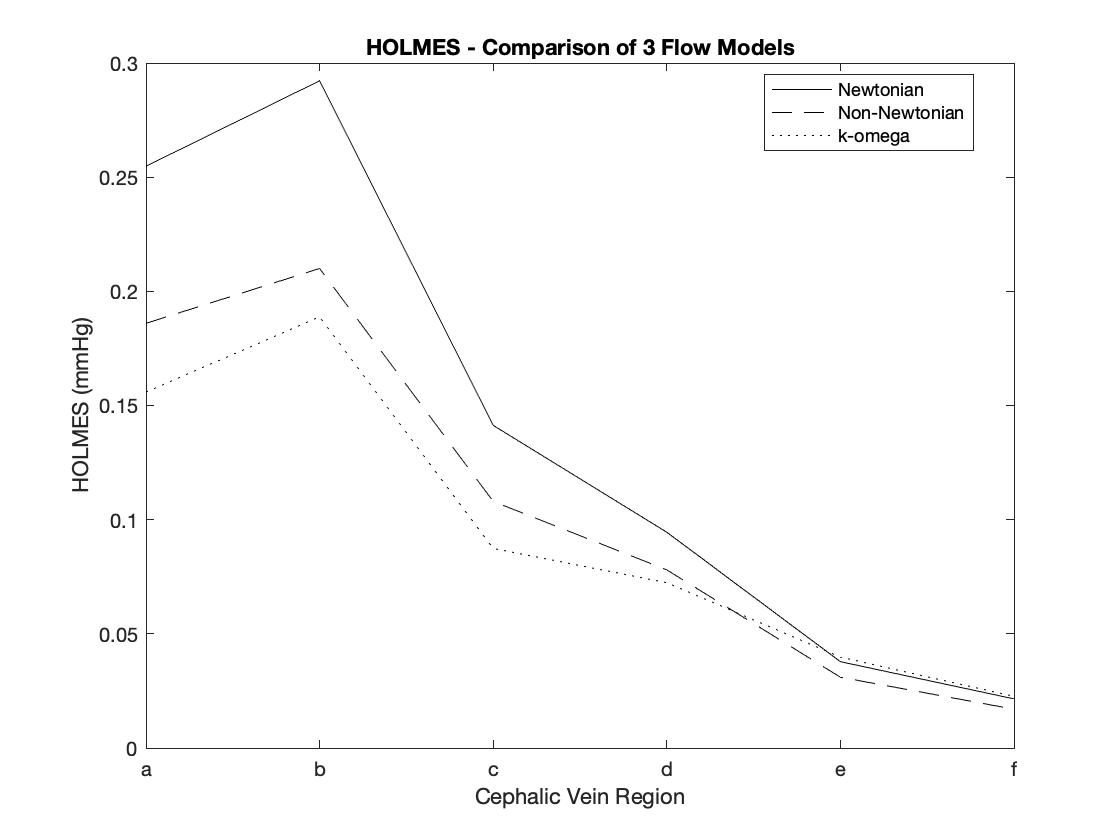
**Comparison of Highly Oscillatory Low MagnitudE Shear (HOLMES) in the cephalic vein based on 3 different simulations**.

If we compare the measurements of average HOLMES obtained from the three flow simulations (Fig. 5), whilst we still see variations in magnitude between the 3 flow models, they converge in regions of low HOLMES, and the overall distribution patterns are very similar. This suggests that this metric is less sensitive in detecting subtle differences in the flow patterns, however the limitation is not with the metric itself, but likely results from our chosen methodology of averaging values over relatively large vessel segments.

### 3.2 Structural Changes

Despite the fact that this AVF was considered fully matured at the start of this study, the cephalic vein underwent significant structural remodelling over the course of the follow up period.

The follow up was stopped after 13 months due to the need for a fistulaplasty to restore diminished flow volumes through the AVF. We decided that following this procedure, further comparisons to our CFD simulation would be potentially misleading, and in the absence of post plasty axial imaging an updated CFD model was not possible.

At the 10-month ultrasound scan, 2 ectatic segments had developed, corresponding to the regular needling sites in the upper half of the forearm. Moderate NIH was observed in the juxta-anastomotic segment of the cephalic vein, becoming increasingly prevalent in the segment of the vein immediately distal to the needling zones. No significant, focal stenotic lesions were identified, but flow volumes were slightly reduced when compared with the baseline ultrasound scan.

The 13-month ultrasound showed progression of NIH in the cephalic vein, resulting in a significant stenosis, extending from 22 mm above the anastomosis up to the level of the ectatic, needling zone. Flow volumes in the feeding artery had decreased from 700 mL/min to approximately 250 mL/min. High recirculation rates were reported on dialysis and the patient was referred for venoplasty of the compromised vessel segment.

### 3.3 Comparison of Haemodynamic Indices and NIH Development

**Fig. 6.**
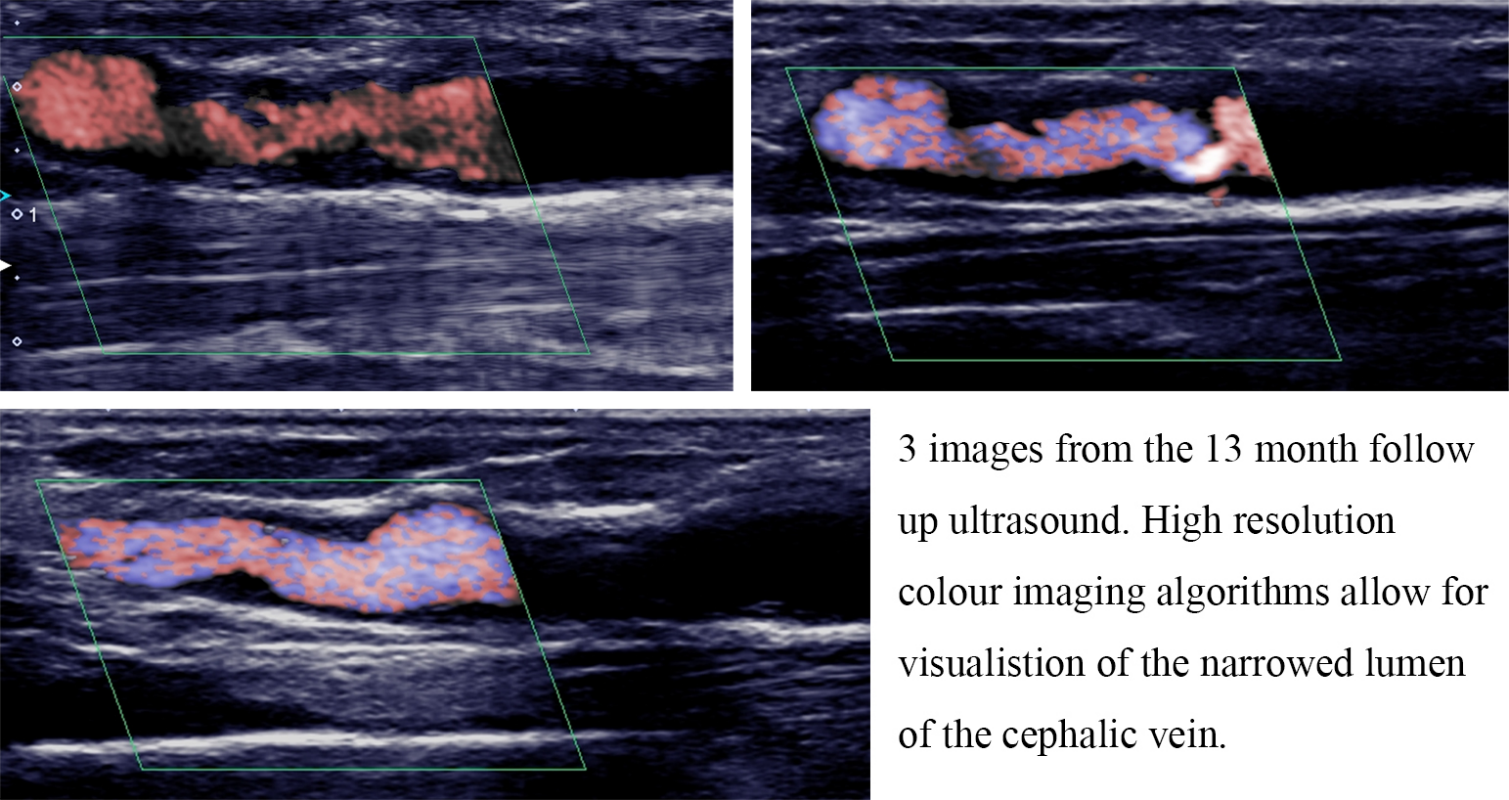
**Ultrasound images showing development of neointimal hyperplasia in the efferent vein**.

Figs. 3,4 show the distribution of OSI and Holmes respectively, whilst the data presented in Table 3 shows the maximum, minimum and average values across the pre-defined regions of the upper limb anatomy (Fig. 1). Fig. 6 shows regions of NIH development, as visualized on the 13-month ultrasound.

**Table 3.** **Shear stress metrics vs neo-intimal hyperplasia (NIH) development in the 10 assessment zones**.

	Zone	USTIR	OSI Max	OSI Ave	HOLMES Ave	WSS Min	WSS Ave	NIH
Vein	a	12.94	0.099	0.012	0.186	0.027	0.237	Minor
	b	6.17	0.460	0.027	0.210	0.009	0.284	Minor
	c	5.55	0.479	0.110	0.108	0.007	0.159	Moderate
	d	10.98	0.496	0.138	0.078	0.002	0.131	Significant
	e	10.43	0.489	0.170	0.031	0.004	0.056	Significant
	f	7.68	0.490	0.162	0.017	0.002	0.026	Significant
Artery	A	8.21	0.437	0.014	0.089	0.012	0.124	Minor
	B	8.21	0.470	0.055	0.056	0.002	0.090	None
	C	8.51	0.475	0.058	0.091	0.005	0.133	None
	D	8.45	0.457	0.060	0.112	0.007	0.163	None

USTIR, ultrasound derived turbulence intensity ratio; OSI, oscillatory shear index; HOLMES, Highly Oscillatory Low MagnitudE Shear; WSS, wall shear stress; NIH, neo-intimal hyperplasia.

In the venous portion of the AVF the segments with moderate or significant NIH development corresponded with the regions of lowest average HOLMES and average WSS values, as well as the highest average OSI. The absolute maximum OSI and minimum HOLMES recorded from each venous segment did not map to the regions of NIH development, but this is not surprising given the non-uniform distribution of WSS throughout the AVF circuit.

No obvious relationship was identified between any of the WSS indices and development of NIH in the arterial segments of the AVF, however given the lack of significant structural remodelling which occurred in the artery, meaningful interpretation of the data obtained from this region of the model is very limited.

### 3.4 Ultrasound Turbulence Intensity Ratio

**Fig. 7.**
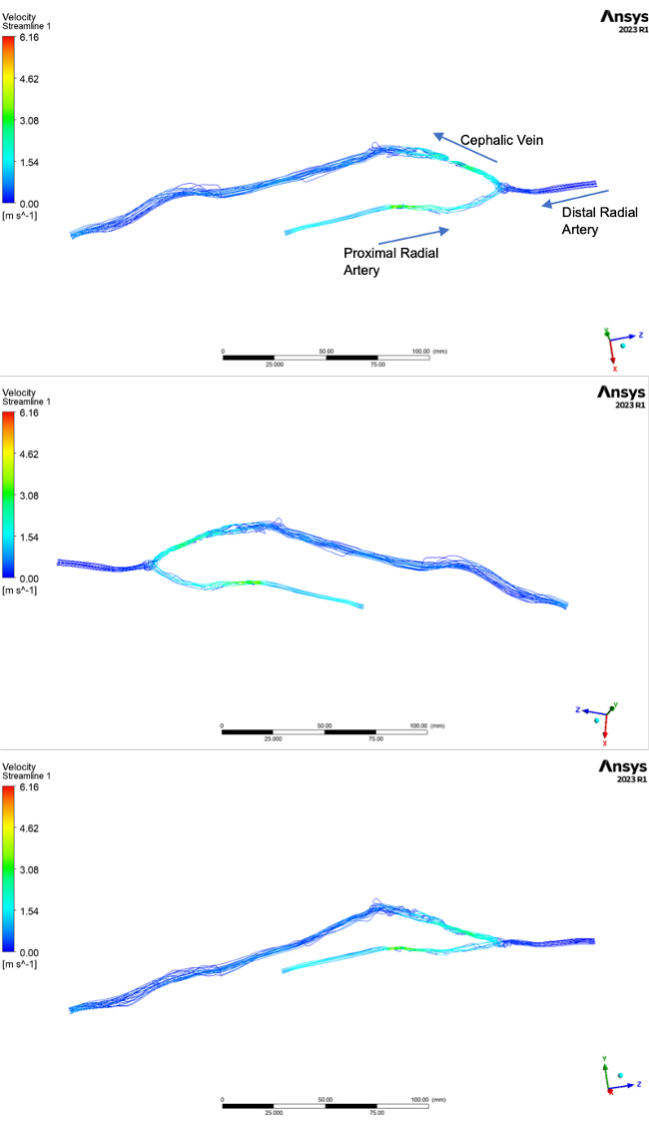
**Streamline plots generated from the CFD simulation**. Note: The side branch is not visible on these images as only 2% of the total flow was diverted into this vein. The geometry used to generate these plots was identical to that represented in Figs. 1,3,4. CFD, computational fluid dynamics.

USTIR is highly elevated at the anastomosis despite the presence of only minor NIH in this region on the 13-month ultrasound, and the comparatively low OSI, and high HOLMES values. This is likely due to the inertial forces resulting from the sudden change in flow direction at the anastomosis leading to an elevated USTIR in the absence of true turbulence. The streamline plot in Fig. 7, shows a helical flow pattern through the anastomotic region, at the point where fluid from the 2 independent inlets mixes.

Beyond the anastomosis USTIR in the vein corresponds well with both the WSS indices and the observed structural remodelling and NIH development.

## 4. Discussion 

The proliferative NIH development observed in this patient is typical of the late onset negative remodelling, which causes successfully matured, previously functional vascular access fistulae to fail. We know that this form of negative luminal remodelling can be triggered in response to a range of haemodynamic, biological and traumatic stimuli, and whilst it would be wrong to infer causality from a single patient case study, the unique dataset available to us regarding the flow parameters of this patient’s AVF provides a useful insight into the haemodynamic conditions surrounding this event.

In the case presented the flow limiting NIH development was not in the most commonly reported juxta-anastomotic segment, but was several centimetres further downstream.

The segments of the efferent vein with the highest USTIR recorded on the initial scan corresponded to the segments of vein, which showed the greatest average OSI values on the CFD model. Both OSI and USTIR have been previously linked with accelerated NIH formation and these associations fit with the distribution of NIH observed in this patient. The distribution patterns of OSI show a non-circumferential pattern, and we acknowledge that by averaging OSI over longitudinal segments of the vessels, information relating to the impact of localised hotspots is lost. This approach was chosen to enable regional comparisons with USTIR values obtained using Doppler ultrasound, which does not offer the temporal and spatial resolution of CFD simulations.

HOLMES is a combined metric for quantifying regions of elevated OSI and low WSS, both of which have previously been used as predictors of NIH development [18]. The average HOLMES value over each segment of the efferent vein corresponded well with the distribution of NIH, with the lowest values being observed in the most severely compromised segments of the vessel. HOLMES appeared less reliable in the feeding artery, with low values observed throughout despite the absence of any significant structural changes. Our methodology of averaging values over pre-defined segments of the vessels is likely to have underestimated the relevance of this metric. Due to the increased flow volumes, and irregular luminal diameters the magnitude of shear forces vary hugely throughout the AVF circuit, and as a result by averaging HOLMES over each segment, we may fail to account for localised extremes of WSS. HOLMES is a valuable metric for use in CFD studies investigating the impact of surgical technique or implantable devices, where localised regions of high OSI and low TAWSS are of great importance, however the limitations associated with averaging HOLMES over larger vessel segments mean that it is difficult to correlate with haemodynamic data from ultrasound, which does not offer the excellent spatial resolution that can be achieved with CFD modelling.

Fig. 7, shows the streamline patterns through the AVF and although quantitative assessment is difficult, it is clear that the segments of the efferent vein where flow is most disturbed were successfully identified on both ultrasound assessment and the CFD model, through elevated USTIR and OSI respectively. The streamline plots also help explain why USTIR measurements made at the anastomosis may be misleading and fail to correspond to NIH formation. The helical flow patterns through the anastomosis and in the juxta-anastomotic vein will result in a dramatic spectral broadening of the Doppler trace, due to the ultrasound system’s assumptions of uni-planar data acquisition via a beam of uniform width. These assumptions likely result in a falsely elevated USTIR resulting from directional variability within the flow field despite the absence of true turbulence. Similarly, the raised USTIR values obtained from the feeding artery are likely due to the effects of vessel curvature and retrograde flow in the distal forearm.

Neointimal hyperplasia can form in response to vascular trauma from surgery, angioplasty or venepuncture. This acute response to injury may partially explain, why some AVF fail to mature, and suffer from accelerated NIH formation and thrombosis in the initial days and weeks following surgical formation. However, this mechanism does not provide an intuitive explanation for the NIH development observed in the case presented above, which occurred in a previously well-functioning AVF, and was seemingly unprovoked by trauma or injury. Nor does it fully explain the most commonly reported distributions of flow limiting NIH; the juxta-anastomotic swing segment, and the cephalic arch.

NIH has been shown to form in regions of high OSI [15], low HOLMES [19] and elevated USTIR [8], observations that are consistent with the case study presented here. Although different, these 3 metrics all correspond to regions of disturbed or turbulent flow, and whilst the exact mechanism responsible for NIH proliferation cannot be confirmed, we can hypothesise that in the presence of disturbed flow, the normal flow mediated functions of the endothelium is compromised.

### Limitations 

The extent of the inward luminal remodelling in this particular case meant that localised regions of elevated OSI could not be directly correlated to regions of smooth muscle cell proliferation. Future studies of this type would benefit from more regular surveillance of the vessel walls to better establish the relationship between elevated OSI and the onset of NIH formation. Although the ethical considerations surrounding most multi-planar image acquisition are a limiting factor in such work, ultrasound imaging at regular intervals may provide a degree of insight into the natural history of NIH progression.

Although care was taken to make the CFD model as robust as possible, a number of assumptions and simplifications were made in order to balance physiological accuracy with computational expense, including rigid vessel walls and laminar flow conditions. It is also important to recognise that the haemodynamic scenario modelled in our simulation corresponds to resting flow conditions at a single time point. During dialysis the position and angle of the needles, along with the pump speed will have a significant impact on the flow through the circuit, resulting in a very different set of haemodynamic conditions, with localised regions of both high and low WSS combined with oscillatory flow patterns [20]. Further to this, flow will likely vary in the periods between dialysis sessions due to filtration induced fluctuations in viscosity, and blood pressure [21].

## 5. Conclusions

This enhanced, computational case study provides further support for the notion of oscillatory shear forces playing a major role in the process of accelerated NIH formation, leading to dialysis access failure. These oscillatory forces can be located and quantified with the use of OSI, as well as other useful metrics such as HOLMES, however these values can only be obtained from complex, computationally expensive CFD models, which are not currently a viable tool for individual patient diagnostics or surveillance. The application of USTIR to the case presented here demonstrates the potential of using Doppler ultrasound to identify regions, which may be prone to these deleterious haemodynamic forces, which we cannot directly measure in the clinical setting. In addition to informing protocol design for further research into the diagnostic power in USTIR in vascular access surveillance, we hope that this work will encourage further collaboration between clinical and engineering staff, and will aid the translation of knowledge obtained from *in-silico* research into tools more suited to point of care testing.

## Data Availability

For enquiries relating to the original dataset please contact the corresponding author.
